# Microsatellite Markers Developed Based on Transcriptomic Data Reveal the Genetic Diversity and Population Genetic Structure of *Angulyagra polyzonata* in Guangxi, China

**DOI:** 10.3390/biology14101424

**Published:** 2025-10-16

**Authors:** Shengjie Zhang, Dapeng Wang, Kangqi Zhou, Yong Lin, Zhong Chen, Junqi Qin, Xuesong Du, Liuping Long, Caiqun Zhang, Xianhui Pan, Wenhong Li

**Affiliations:** 1 College of Animal Science and Technology, Guangxi University, Nanning 530003, China; 17860361055@163.com; 2 Guangxi Key Laboratory of Aquaculture Genetics and Breeding, Guangxi Academy of Fishery Sciences, Nanning 530021, China; oucwdp@163.com (D.W.); zhoukqfisher@163.com (K.Z.); linnn2005@126.com (Y.L.); evzccjy0824@126.com (Z.C.); 19118780443@163.com (J.Q.); gslnlxr@163.com (X.D.); 19100243093@163.com (L.L.); zcqunwy@126.com (C.Z.)

**Keywords:** *Angulyagra polyzonata*, nucleotide repeat sequence, microsatellite markers, genetic diversity

## Abstract

**Simple Summary:**

This study focused on *Angulyagra polyzonata*, an economically important freshwater snail in Guangxi, China, whose wild populations have declined sharply due to overharvesting. To assess its genetic status, we developed nine novel microsatellite markers via transcriptomic analysis following the screening of a total of 798,244 SSR loci. These markers were then used to analyze 360 individuals from 12 wild populations across the region. This study provides crucial baseline data for conserving *A. polyzonata* and highlights the value of integrating whole-genome data into future research to refine management strategies. Additionally, the developed microsatellite markers represent valuable tools for the ongoing monitoring of this ecologically and economically important species.

**Abstract:**

*Angulyagra polyzonata* is a significant freshwater snail species in southern China. However, its wild resources have sharply declined due to overfishing. To assess the current status of germplasm resources in the Guangxi region, during this study, we first successfully developed nine pairs of primers that enable the amplification of highly polymorphic microsatellite markers (SSRs) with trinucleotide and tetranucleotide repeat sequences (PIC values ranging from 0.662 to 0.861) using transcriptomic data. Then, these designed primers were tested and applied for the genetic investigation of selected wild populations of the species. Finally, a genetic diversity analysis was conducted based on 12 wild populations (360 individuals) in Guangxi. After 798,244 SSR loci were screened out via high-throughput sequencing, the results showed that dinucleotide repeats accounted for the highest proportion (47.64%), mainly consisting of (AC/GT)n repeat units. Among the SSR loci in *A. polyzonata*, microsatellite loci with 5 to 20+ repeats are the most abundant. All nine selected and tested SSR loci significantly deviated from Hardy–Weinberg equilibrium (*p* < 0.001) and had heterozygote deficiency (average inbreeding coefficient of *F* = 0.390), indicating widespread inbreeding. The fixation index among populations was high (average *Fst* = 0.175), with 73% of the genetic variation occurring within populations and 27% between populations. Gene flow (*Nm*) was generally restricted (most population pairs had *Nm* < 1), with the (Tiandeng) TD and (Long’an) LA populations showing the smallest differentiation (*Fst* = 0.017), and the (Qinnan) QN and (Yinhai) YH populations showing the greatest differentiation (*Fst* = 0.409). UPGMA clustering and structure analysis (K = 2) divided the 12 populations into two subgroups. Overall, our research suggests that the genetic diversity of the wild population of *A. polyzonata* in the Guangxi region has declined. Thus, prioritizing the protection of highly genetically diverse populations, such as the LA population, is urgently needed. This study provides a scientific basis for the protection and sustainable utilization of *A. polyzonata* resources in Guangxi.

## 1. Introduction

*Angulyagra polyzonata*, a significant freshwater snail species belonging to the Gasteropoda class, Viviparidae family, and *Angulyagra* genus, is widely distributed across Southern China and various Southeast Asian countries [1]. In China, it can be found in freshwater lakes, rivers, streams, and ditches. Renowned for its high nutritional value and distinct flavor [2], this snail has long been a favorite among consumers. Moreover, its appealing appearance and distinct edge patterns have recently propelled it to emerge as a rising star in the ornamental market. In recent years, Liuzhou Luosifen from Guangxi has gained global popularity, standing out as one of China’s geographically representative products [3]. In 2024, the total output value of the Luosifen industry exceeded RMB 75 billion. As a core raw material in this booming industry, the market demand for snails is enormous, with annual consumption surpassing 1.5 million tons. However, China’s Viviparidae economic freshwater snail breeding output is only approximately 96,900 tons, leaving the vast majority to be sourced through wild fishing [4]. This heavy reliance on wild populations, coupled with the impacts of human activities, has significantly affected the population size of *A. polyzonata* in the wild. Under such circumstances, establishing a method to assess the current status of genetic resources in China’s *A. polyzonata* populations has become a critical and urgent task.

Microsatellite markers, also known as simple sequence repeats (SSRs), are widely used in population genetic analyses due to their convenience and practicality [5,6]. The currently commonly used methods for developing microsatellite markers are categorized into five types: traditional gene library construction, microsatellite enrichment, homologous transfer, public database search, and transcriptome sequencing methods [7]. Broadly speaking, the transcriptome refers to the collection of all RNAs transcribed within a cell under a specific condition; narrowly speaking, the transcriptome typically refers to the collection of all RNA transcripts. The research methods currently used for determination of the transcriptome mainly fall into two categories: one is based on hybridization techniques, including microarray technology and gene chip technology; the other is primarily based on sequencing technology, including expression sequence tag technology, gene expression series analysis technology, and RNA sequencing technology [8]. RNA sequencing (RNA-seq) is a typical representative of “next-generation” sequencing technology, which can sequence millions of DNA or RNA molecules at once [9]. Compared to other sequencing technologies, RNA-seq offers several advantages, including high throughput, high resolution, high sensitivity, no species restrictions, a wide dynamic range, and good repeatability. Simultaneously, this sequencing technology can also identify positional genes and discover new transcripts in the species [10]. Since the advent of microsatellite markers, their application has extended to various freshwater snail species, including *Cipangopaludina chinensis* [4], *C. cathayensis* [11], *Bellamya purificata* [12,13], and *Promenetus exacuous* [14].

Current research on *A. polyzonata* primarily focuses on its nutritional components [2], its role in parasite transmission [15], and its habitat distribution [16]. In genomic studies, Zhang and others assembled the mitochondrial genome structure of *A. polyzonata* using second- and third-generation sequencing while exploring its phylogenetic relationships within the Viviparidae family [17]. Additionally, Zhu and others analyzed SSR molecular markers in *A. polyzonata* from Hunan, China, using the Microsatellite Identification Tool (MISA), revealing high heterozygosity and a low number of repetitive sequences in the species [18]. However, investigations into the population genetic diversity and genetic structure of *A. polyzonata* remain relatively limited. In this study, we employed the RNA-seq transcriptome method to design nine pairs of specific microsatellite primers for *A. polyzonata* in the Guangxi region. The findings are expected to provide new insights for the conservation of wild germplasm resources and artificial selective breeding of *A. polyzonata*.

## 2. Materials and Methods

### 2.1. Experimental Materials and DNA Extraction

**Figure 1:**
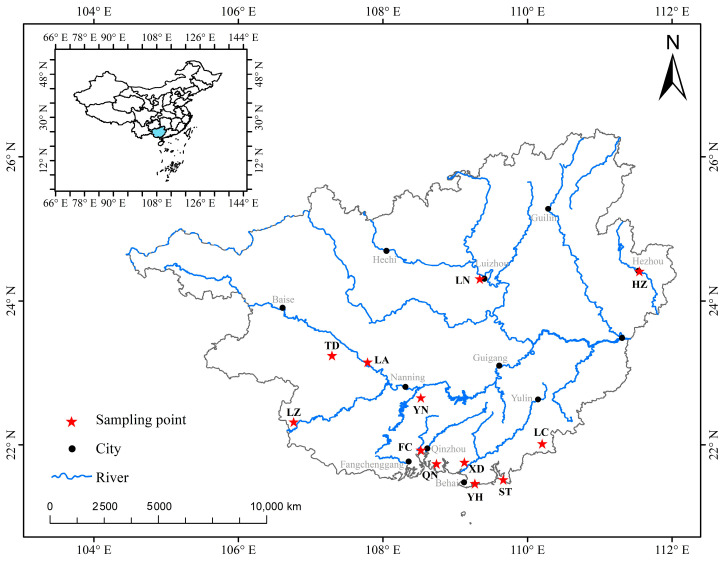
Sampling distribution map of *Angulyagra polyzonata* in the Guangxi region. Note: The blue area in the upper left corner of the picture represents Guangxi Zhuang Autonomous Region of China.

The materials used for the experiment were sourced from 360 wild *A. polyzonata* specimens collected across 12 regions of Guangxi between June and July 2024 (Figure S1). The specific locations included Yongning (YN), Tiandeng (TD), Long’an (LA), Longzhou (LZ), Luchuan (LC), Fangcheng (FC), Qinnan (QN), Yinhai (YH), Xingdao (XD), Shatian (ST), Hezhou (HZ), and Liunan (LN). Thirty samples were taken from each location. Details, including the latitude and longitude of sampling sites, as well as the number of individuals, are presented in Figure 1 and Table 1.

All samples were transported under low-temperature conditions to the Guangxi Academy of Fishery Sciences, where they were temporarily housed in stepped tanks to enable the recovery of activity. Following anesthesia with MS-222, the snails were dissected (approved by the Guangxi Institutional Animal Care and Use Committee (GACUC number 201703021; date: 30 September 2024), and fresh muscle tissue was harvested, placed into 1.5 mL centrifuge tubes, and preserved in an appropriate volume of 95% anhydrous ethanol at −20 °C in a medical refrigerator. Genomic DNA of *A. polyzonata* was extracted using the Omega Animal DNA Extraction Kit (REF: D3396-02, Omega Bio-Tek Inc., Norcross, GA, USA). The quality and integrity of extracted DNA were assessed via 1% agarose gel electrophoresis, while its concentration and purity were determined using a NanoDrop ONE spectrophotometer (ThermoFisher, Waltham, MA, USA).

### 2.2. Library Construction and SSR Search

One DNA sample was selected from each of three sampling sites: Fangcheng (FC), Yinhai (YH), and Shatian (ST). Libraries with an insert fragment size of 400 bp were constructed for each sample, followed by paired-end (PE) sequencing using the Illumina NovaSeq platform based on next-generation sequencing (NGS) technology. Initial sequencing data statistics are detailed in Table S1. Raw sequencing data were filtered using fastp (v0.20.0; https://github.com/OpenGene/fastp, accessed on 15 June 2025) to remove 3′ end adapter contamination and retain high-quality sequences [19]. Quality filtering employed a sliding window approach with a 5 bp window, which was slid from the 3′ end to the 5′ end to calculate base quality (Q) values. Bases within the current window were truncated if the Q value was below 20; otherwise, sliding was terminated, and PE reads were retained or discarded based on their length.

Given the PE sequencing mode, data from all samples were merged into a combined dataset (designated as popA). High-quality reads were then obtained by overlapping PE sequences using FLASH (v1.2.11; https://ccb.jhu.edu/software/FLASH/, accessed on 16 June 2025) [20] with the following parameters: minimum overlap length = 10 bp, maximum mismatch density = 0.2, and “outie” pair allowance = false. Detailed information for popA is provided in Table S2. Microsatellite (SSR) loci were identified using MISA (Microsatellite Identification Tool; http://pgrc.ipk-gatersleben.de/misa/, accessed on 17 June 2025) [21] with the following parameters: ≥10 mononucleotide repeats; ≥6 dinucleotide repeats; and ≥5 tri-, tetra-, penta-, and hexanucleotide repeats. The maximum interval between two SSRs was set to 100 bp, and reverse complement sequences as well as shifted permutations were treated as identical SSR types.

### 2.3. SSR Clustering and Polymorphism Assessment

Repetitive sequences in the sequences were masked using the Perl program (replaced with the letter “R”), and SSRs with flanking sequences shorter than 20 bp were filtered out. The filtered sequences were clustered using cd-hit (v4.5.7; https://github.com/weizhongli/cdhit, accessed on 18 June 2025) [22] with the following parameters: nucleotide sequence similarity set to 90%, coverage to 70%, and gap penalties specified as -gap 1 -gap-ext 0. Sequences containing two or more SSRs were counted and clustered separately. The statistical results of SSR clustering are presented in Table S3. Clustering results were further analyzed using the Perl program, with each cluster categorized based on the length of the SSR. Polymorphism of each cluster was determined as follows: a polymorphism value of 1 was assigned if all SSRs in the same cluster had identical lengths, a value of 2 if two distinct lengths were present, etc. The statistical results of SSR polymorphism for each cluster are shown in Table S4.

### 2.4. Design of SSR Primers

Primer3 (version 2.3.6; https://sourceforge.net/projects/primer3/files/primer3/2.3.6/, accessed on 20 June 2025) [23] was used to design primers for SSR sequences within clusters with polymorphism values > 2, with primer binding sites located at both ends of the sequences. The 5′ end of the upstream primer was modified to include the M13 universal primer sequence (TGTAAAACGACGGCCAGT), and M13 primer sequences labeled with different fluorescent groups were synthesized. The length of the target amplified fragment was controlled within 100–400 bp, and the amplification range was set from the first base upstream of the repetitive sequence to the fifth base downstream of the repetitive sequence. A total of 144 pairs of primers were generated; primers with flanking sequence lengths less than 20 bp and target amplification fragment lengths exceeding the range of 100–400 bp were excluded. Through evaluation using the Primer3 software (version 2.3.6), primers with a high probability of self-complementary sequences were excluded. Initially, 33 pairs of primers with potential polymorphisms were selected.

### 2.5. Verification of SSR Loci and Screening of Polymorphisms

**Figure 2:**
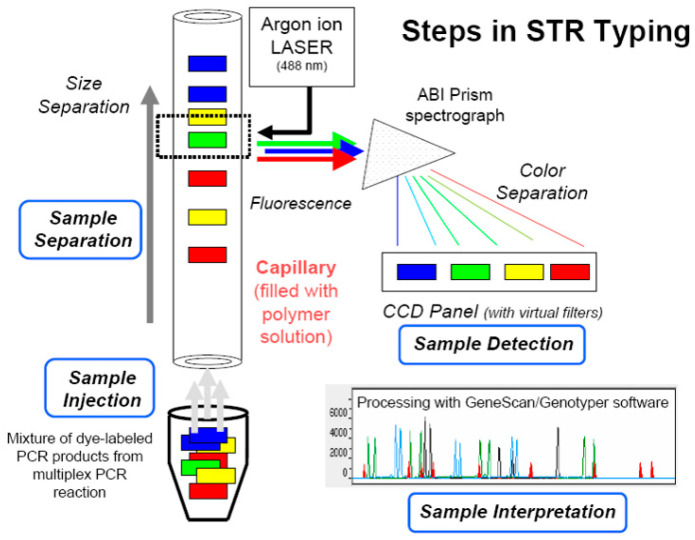
A schematic diagram of the principle of fluorescence capillary electrophoresis typing. Note: STR is the abbreviation for Short Tandem Repeat.

To validate the SSR loci and screen for polymorphic markers, the 12 aforementioned geographical populations of *A. polyzonata* from Guangxi were used. The simplex PCR strategy was employed for the amplification of the 9 loci, meaning that each reaction system specifically amplified only 1 locus. The reaction was performed on a Veriti 384 PCR instrument (Applied Biosystems, Waltham, MA, USA) with the following program: label each pair of forward primers at its 5′ end with a fluorescent dye (TAMRA, HEX, ROX, or FAM). First, add 5.0 μL of 2× Taq PCR Master Mix reagent and 1.0 μL of DNA, and perform a 5-min pre-denaturation treatment at 95 °C. Then, perform a 30-s denaturation treatment, a 30-s gradient annealing (between 62 and 52 °C), and extend for 30 s at 72 °C, running 10 cycles; add 0.5 μL of the upstream and downstream primers (concentration 10 pmol/μL), and add 3.0 μL of ddH_2_O. Perform a 30-s denaturation treatment at 95 °C, a 30-s annealing treatment at 52 °C, and a 30-s extension treatment at 72 °C. Run 25 cycles, extend at 72 °C for 20 min, and finally store at 4 °C. Each individual’s DNA sample undergoes 9 independent single-round PCR reactions, resulting in amplification products at 9 corresponding loci. After all the amplifications are completed, the 9 amplified products from the same individual are mixed at equal molar concentrations in a single centrifuge tube. After PCR completion, the amplified products were analyzed using fluorescence capillary electrophoresis (Figure 2).

### 2.6. Data Processing and Analysis

Raw data were acquired from the ABI 3730xl platform and exported as .fsa files. After classification by locus, the data were imported into GeneMarker (v3.0.0; https://softgenetics.com/products/genemarker/, accessed on 21 June 2025) [24] to generate and export Excel-formatted genotype data and PDF files of genotyping peak profiles.

Genetic diversity indices for both SSR loci and populations were calculated using GenAlEx (v6.501; https://biology-assets.anu.edu.au/GenAlEx/Welcome.html, accessed on 23 June 2025) [25], including the observed number of alleles (*Na*), the effective number of alleles (*Ne*), Shannon’s information index (*I*), polymorphism information content (*PIC*), observed heterozygosity (*Ho*), expected heterozygosity (*He*), and inbreeding coefficient (*F*). The inbreeding coefficient was computed using the formula *F* = 1 − *Ho*/*He*.

Genetic distances between populations were calculated with PowerMarker (v3.25; https://en.freedownloadmanager.org/Windows-PC/PowerMarker-FREE.html, accessed on 25 June 2025) [26]. In the PowerMarker software, cluster analysis is conducted using the unweighted pair-group method with arithmetic mean (UPGMA) based on Nei’s genetic distance, and a tree diagram is generated.

Population structure of the 360 samples was analyzed using STRUCTURE (v2.3.4; https://web.stanford.edu/group/pritchardlab/structure_software/release_versions/v2.3.4/html/structure.html, accessed on 26 June 2025). The parameter K (number of hypothetical populations) was set from 1 to 20, with a burn-in period of 10,000 and 100,000 Markov Chain Monte Carlo (MCMC) iterations. Each K value was run 20 times, and the optimal ΔK value (indicating the best population stratification) was determined [27]. Visualization was generated based on results from the optimal K.

Based on population genetic structure analyses, GenAlEx was further used to assess genetic variation and differentiation within and between populations. The fixation index (*Fst*) and gene flow (*Nm*) were calculated, with gene flow determined by the formula *Nm* = 0.25(1 − *Fst*)/*Fst*.

## 3. Results

### 3.1. The Number and Distribution of SSR Loci

Using the MISA software, we successfully obtained the simple sequence repeats from the sample popA of *A. polyzonata*. Among 664,946 sequences, 798,244 SSR loci were found. The occurrence frequency (the proportion of sequences containing SSR among the total sequences) was 9.44%. Among SSR loci, there were 126,494 compound SSR loci, accounting for 15.85% of the total SSR loci. The frequency of SSR loci occurrence (the frequency of SSR loci appearing in the total sequence) was 11.33%. Among 430,110 sequences, 64.68% contained a single SSR locus. Additionally, 108,342 sequences contained more than one SSR locus, accounting for 16.29% (Table 2).

### 3.2. SSR Repetitive Type and Characteristics

The SSR repeat types in *A. polyzonata* exhibit considerable diversity, encompassing one to six nucleotide repeat motifs, though the number of SSR loci varies significantly across different nucleotide repeat types. Among these, dinucleotide repeats are the most prevalent, accounting for 47.64% of the total, followed by mononucleotide repeats at 33.34%. Tetranucleotide and trinucleotide repeats constitute 9.42% and 9.36%, respectively, while pentanucleotide and hexanucleotide repeats are relatively rare, representing only 0.20% and 0.04% of the total SSR loci (Table 3).

**Figure 3:**
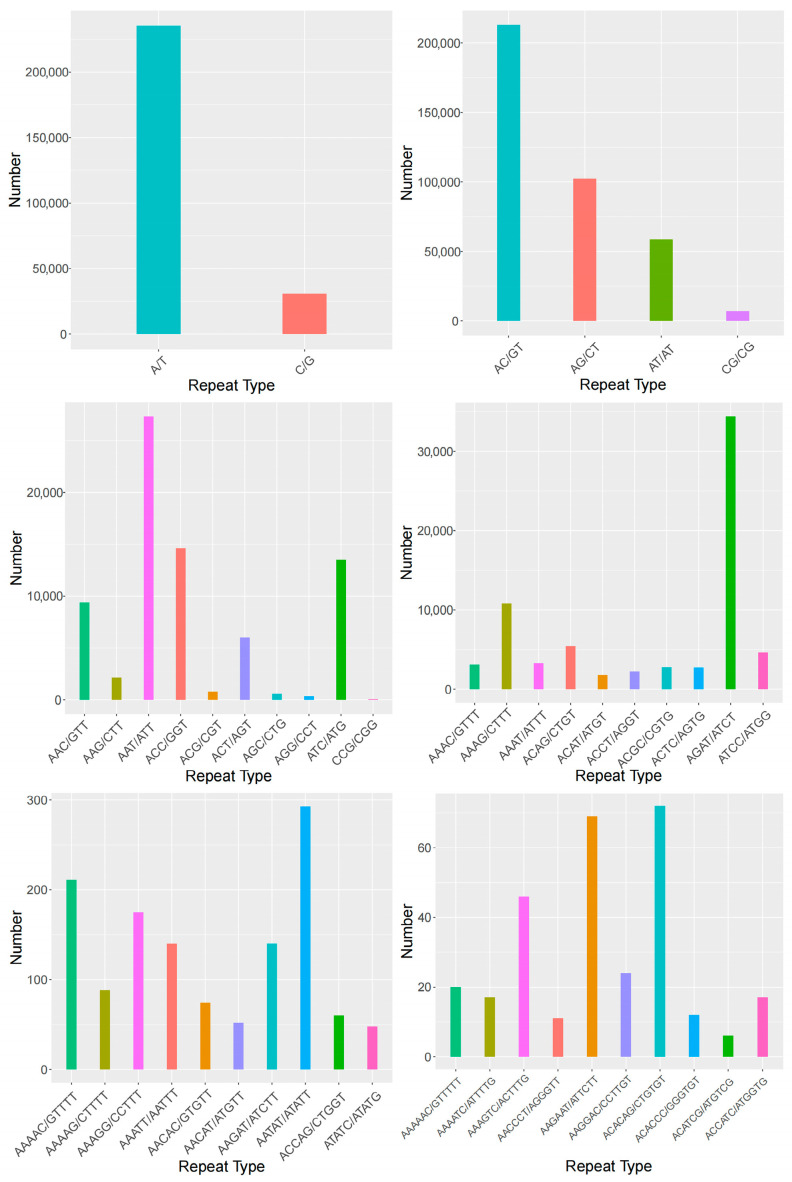
Statistics of SSR motif quantity (this figure presents the statistical results of single, double, and triple nucleotide repeat motifs, as well as the top ten statistical results of the four to six-nucleotide repeat motifs).

In terms of the repeat units within *A. polyzonata* SSR sequences, mononucleotide repeats are dominated by (A/T)n, with 235,187 loci accounting for 29.46% of the total. Dinucleotide repeats are primarily (AC/GT)n, comprising 212,705 loci (26.65% of the total). Trinucleotide repeats are mostly (AAT/ATT)n, with 27,379 loci making up 3.42%. Tetranucleotide repeats are predominantly (AGAT/ATCT)n, totaling 34,416 loci (4.31%). Pentanucleotide repeats are mainly (AATAT/ATATT)n, with 293 loci (0.04%). Hexanucleotide repeats are dominated by two motifs: (AAGAAT/ATTCTT)n and (ACACAG/CTGTGT)n, containing 69 and 72 loci, respectively, each accounting for approximately 0.009% of the total SSR loci (Figure 3).

Among the SSR loci in *A. polyzonata*, microsatellite loci with 5 to 20+ repeats are the most abundant. With the exception of mononucleotide repeats (which have a minimum of 10 repeats) and hexanucleotide repeats (which are mostly in the 5–7 repeat range), dinucleotide, trinucleotide, tetranucleotide, and pentanucleotide repeats are predominantly distributed in the 5–20+ repeat range, with dinucleotide repeats showing the highest distribution frequency (Table 4).

### 3.3. Primer Polymorphism Analysis

**Figure 4:**
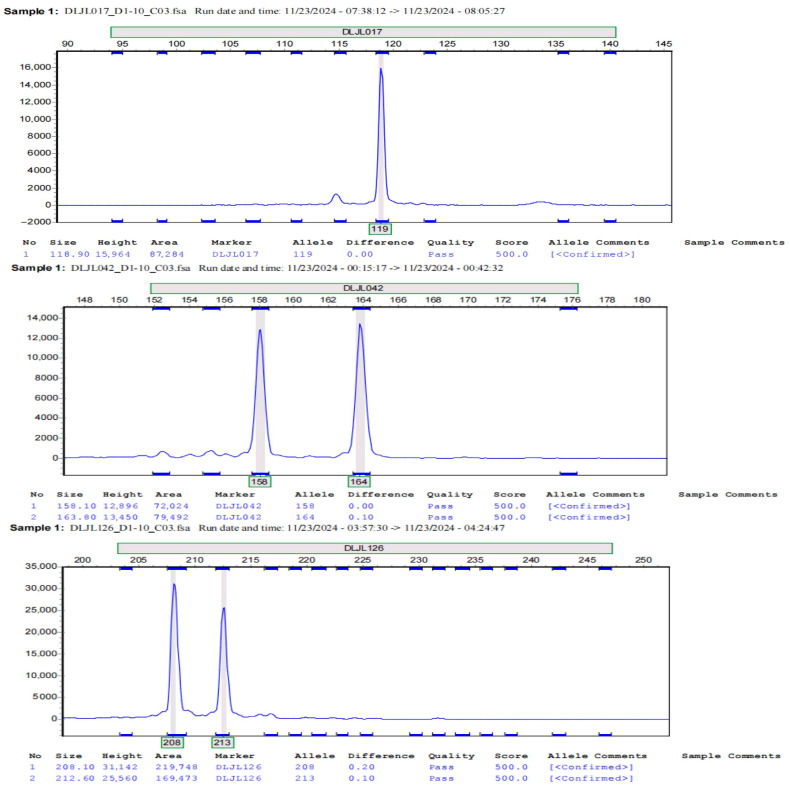
Fluorescence capillary electrophoresis of partial SSR primer amplification.

Using a mixture of DNA from 12 different geographical populations of *A. polyzonata* as the template (performing operations of equal-sized mixing within the same population and equal-proportion merging between different populations for the extracted DNA), 33 primers were subjected to PCR amplification, and the amplification products were analyzed using fluorescence capillary electrophoresis (Figure 4). Primers with low peak intensity, non-target interferences, and overlapping peaks were excluded. Eventually, nine pairs of SSR primers with high polymorphism and stability were selected (Table 5).

Among 360 *A. polyzonata* samples, the 9 primer pairs detected a total of 119 observed alleles (*Na*), with an average of 13.222 alleles per locus. The effective number of alleles (*Ne*) ranged from 3.388 to 7.856, with a mean value of 5.131. Shannon’s information index (*I*) varied between 1.391 and 2.371, averaging 1.867. For the nine loci, the observed heterozygosity (*Ho*) ranged from 0.356 to 0.598 (mean = 0.480), while the expected heterozygosity (*He*) spanned 0.705 to 0.873 (mean = 0.787). The polymorphism information content (*PIC*) values ranged from 0.662 to 0.861, with an average of 0.761, indicating that all nine selected SSR loci possess high polymorphism (*PIC* > 0.500). The inbreeding coefficient (*F*) varied from 0.310 to 0.519, with a mean of 0.390, suggesting a deficiency of heterozygotes at these loci. Hardy–Weinberg equilibrium tests revealed that all nine loci significantly deviated from Hardy–Weinberg equilibrium (*p* < 0.001) (Table 6).

### 3.4. Genetic Diversity Analysis

Quantitative parameters of genetic diversity among the 12 *A. polyzonata* populations are summarized in Table 7. Regarding the average number of observed alleles per population, the LA population exhibited the highest value (6.667), whereas the FC population showed the lowest (3.000). Similarly, the LA population had the highest average effective number of alleles (4.405), and the YH population displayed the lowest number of alleles (1.663). These two parameters collectively indicate that the LA population has the highest level of genetic variation. The average observed heterozygosity across populations ranged from 0.285 to 0.635, with the ST population achieving the highest value (0.635) and the YH population achieving the lowest value (0.285). Regarding average expected heterozygosity, values spanned from 0.299 to 0.749; the LA population ranked highest (0.749), while the YH population was again the lowest (0.299). The average inbreeding coefficient was 0.148, with the LA population having the highest value (0.311) and the QN population having the lowest value (0.028). These results suggest that the ST and TD populations exhibit the highest heterozygosity and genetic variation, whereas the QN and YH populations show the opposite trend. The ranking of the average Shannon index was consistent with that of the average effective number of alleles, further confirming that the LA population has the highest level of genetic diversity among all populations, while the YH population has the lowest.

### 3.5. Genetic Differentiation

As shown in Table 8, the *Nm* values among the 12 *A. polyzonata* populations ranged from 0.361 to 14.456. Among them, the levels of gene exchange were relatively high between the TD and LA populations (14.456) and between the LZ and LC populations (11.655). In contrast, the gene flow between the QN and YH populations, as well as most other populations, was relatively low, with average values of 0.246 and 0.268, respectively. The fixation index (*Fst*) ranged from 0.017 to 0.409. The smallest genetic differentiation was observed between the TD and LA populations (0.017), while the largest was found between the QN and YH populations (0.409). The degree of genetic differentiation among groups was relatively high (the *Fst* value averaged 0.179). The results of the molecular variance analysis (AMOVA) revealed that genetic variation between populations accounted for 27% of the total, whereas that within populations accounted for 73% (Table 9), indicating that the total genetic variation across all populations is primarily driven by variation within populations. The genetic distances among the 12 populations ranged from 0.145 to 0.733. The closest genetic distance was between the LZ and LC populations (0.145), and the farthest was between the YH and LZ populations (0.733) (Table 10).

### 3.6. Population Genetic Structure

**Figure 5:**
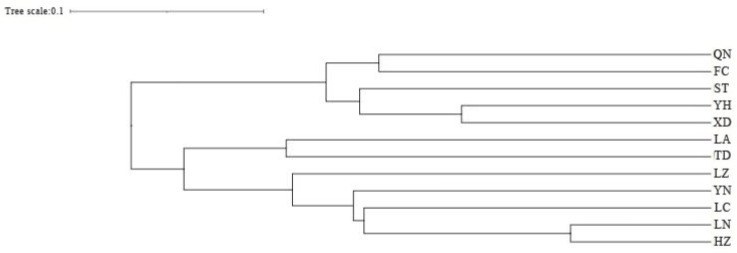
UPGMA clustering results of 12 populations of *Angulyagra polyzonata*.

**Figure 6:**
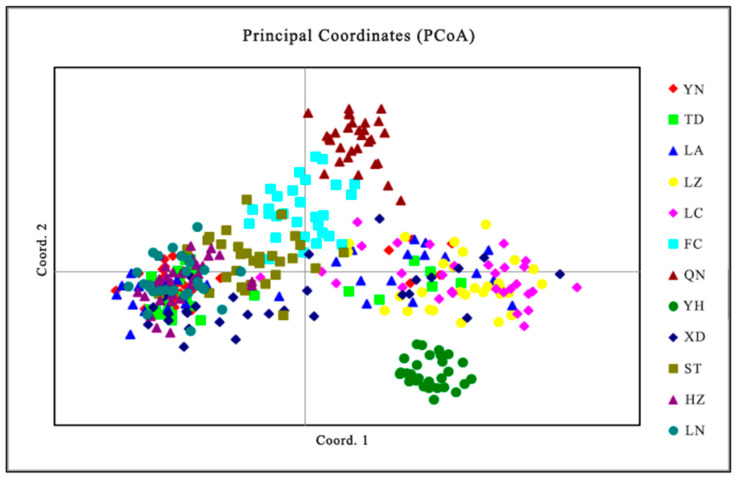
PCoA results for 12 populations of *Angulyagra polyzonata*.

**Figure 7:**
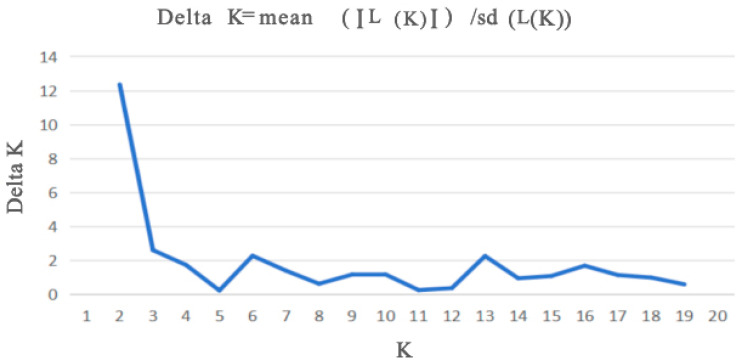
Changes in K value plotted using the ΔK method of structure analysis.

**Figure 8:**
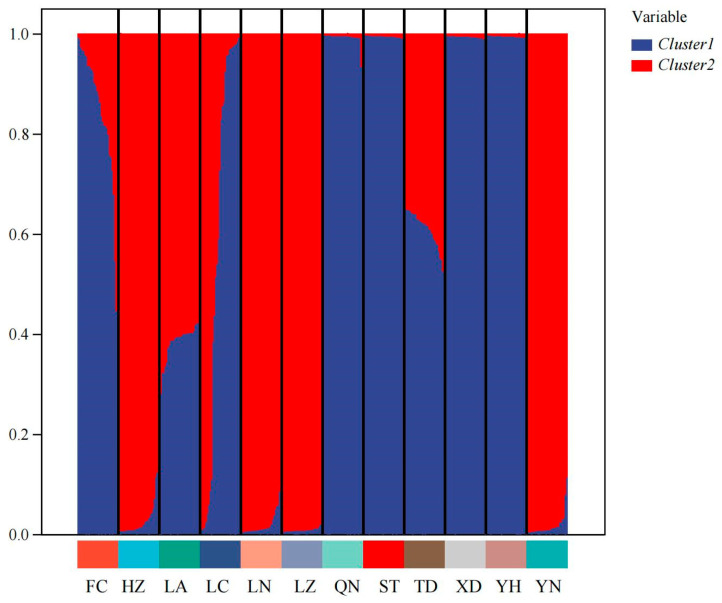
Structure analysis for 12 populations of *Angulyagra polyzonata* (K = 2).

Cluster analysis results showed that the QN and FC populations, as well as the YH and XD populations, initially formed independent clusters, which then merged with the ST population. In addition, the TD and LA populations clustered together, while the HZ and LN populations remained independent initially before merging with the LC and YN populations (Figure 5). As determined from the PCoA results (Figure 6), the YH and QN populations have relatively independent genetic characteristics and are quite distinct from other groups. The LN, HZ, YN, and TD populations are clustered together, while the remaining population sample points are relatively scattered, with many overlapping areas between different populations. Structural analysis of 360 samples using nine microsatellite markers revealed that the optimal K value was 2 (Figure 7), suggesting that the snail populations in Guangxi could be divided into two subpopulations (Table S5, Figure 8 and Figure S2).

## 4. Discussion

In recent years, with the continuous advancement of molecular research techniques, high-throughput sequencing has been widely applied to identify polymorphic SSR loci. In this study, using next-generation sequencing (NGS) based on the Illumina NovaSeq platform, a total of 798,244 SSR loci were identified from 664,946 sequences, with an occurrence frequency of 9.44%. This frequency is lower than that reported for *C. cathayensis* (23.92%), *B. aeruginosa* (13.77%), *V. tricinctus* (23.46%) [18], and *C. chinensis* (20.12%) [28], but slightly higher than that of *Babylonia lutosa* (6.86%) [29] and *Thais luteostoma* (6.45%) [30]. Variations in SSR occurrence frequency are attributed not only to species differences and sampling locations, but also to factors such as sequencing platforms, SSR mining tools, search criteria, and database richness [31,32,33]. In terms of SSR repeat types, *A. polyzonata* is dominated by dinucleotide repeats, accounting for 47.64%. This is consistent with findings in *C. cathayensis* (47.50%), B. *aeruginosa* (48.40%), *V. tricinctus* (50.40%) [18], *C. chinensis* (57.67%) [28], and *Scapharca subcrenata* (58.06%) [34], but differs from *Potamocorbula ustulata* (the mononucleotide repeats accounted for the highest proportion, with a value of 47.64%, and the same applies below) [35] and *S. subcrenata* (33.89%) [36]. Such differences may be related to species specificity, locus mutation rates, and selective evolutionary mechanisms [28,37]. Regarding SSR repeat units, *A. polyzonata* is primarily characterized by (AC/GT)n, which accounts for 29.46% of the total SSR loci. This aligns with previous studies on the SSR repeat sequence characteristics of *A. polyzonata* and four other gastropod species in Hunan Province, China [18]. The dinucleotide repeat sequences of *A. polyzonata* have the highest number of SSR loci, and the repetition frequency is primarily within the range of 6 to 9. This differs slightly from the repeat counts reported for *C. chinensis* (4~7 repeats) [28] and *Hemifusus termatamus* (7~30 repeats) [38]. Thus, the pattern of SSR repeat counts may be influenced by differences in the coding and non-coding regions of species [39]. Currently, most microsatellite marker studies focus on dinucleotide repeats; however, the small differences between alleles often lead to severe peak interference. In contrast, the nine SSR loci selected in this study include five tetranucleotide repeats and four trinucleotide repeats, which can effectively maintain locus polymorphism while improving genetic stability and the accuracy of result resolution [36,40,41].

At present, the polymorphic information content (*PIC*) is widely employed to evaluate the capacity of microsatellite markers in detecting population polymorphism [42]. In the present study, the *PIC* values of 9 microsatellite loci across 12 populations of *A. polyzonata* ranged from 0.662 to 0.861. This indicates that the developed microsatellite loci exhibit high polymorphism (*PIC* > 0.5) and are capable of accurately assessing genetic differences among populations [43]. The inbreeding coefficient (*F*) serves as a tool to measure the deviation between the observed heterozygosity and the expected heterozygosity within a population [44,45]. In this study, the F values for each locus in *A. polyzonata* varied from 0.310 to 0.519, suggesting a deficiency of heterozygotes (*F* > 0) among the 12 *A. polyzonata* populations. This phenomenon may be attributed to inbreeding within these populations. Notably, all nine loci of *A. polyzonata* showed deviations from Hardy–Weinberg equilibrium. Such deviations could arise from factors including inbreeding, natural selection, and the presence of null alleles [46]. Similar deviations of microsatellite loci from Hardy–Weinberg equilibrium have also been reported in *C. cathayensis* [11] and *B. purificata* [47].

The genetic diversity of a species is the result of the evolutionary process of the previous generations. To a certain extent, the adaptability of species to their environment is positively correlated with genetic diversity; specifically, higher genetic diversity enables species to adapt more readily to complex environmental changes [48]. Compared with fish, shrimp, and crabs, the protection, evaluation, and development of snail germplasm resources in China have received relatively less attention. Thus, further research on the genetic diversity of mussel populations such as *A. polyzonata* is essential for understanding the current status of mussel resources in Guangxi. Microsatellite markers are commonly used to assess the genetic diversity of organisms by examining parameters such as the number of alleles (*Na*), the effective number of alleles (*Ne*), Shannon’s information index (*I*), observed heterozygosity (*Ho*), and expected heterozygosity (*He*) [49]. The discrepancy between *Ne* and *Na* reflects the uniformity of allele distribution within a population: a larger difference indicates a more uneven distribution of allele frequencies, with a small number of alleles occurring at high frequencies. Conversely, a smaller discrepancy indicates a more uniform distribution of allele [50]. In this study, when the number of alleles (*Na*) exceeds the effective number of alleles (*Ne*), it indicates that the distribution of alleles within the population of this species is uneven. Furthermore, the highest *Na* was in LA and the lowest in FC, yet FC is surrounded by many other nearby sampling locations (QN, XD, and YH), and the nearest location to LA is TD. This situation may occur because the two populations do not share the same water system. Here, LA belongs to the Xijiang River system, while FC belongs to the system that flows directly into the sea. The *Na* level in the FC population is low. This might be due to the invasion of alien species and habitat destruction, which leads to a reduction in the habitat, a decrease in the size of the native species population, intensified inbreeding, and ultimately, the loss of alleles [4,12]. Observed heterozygosity (*Ho*) and expected heterozygosity (*He*) can be used to quantify the deviation of a population’s actual heterozygosity from its theoretical state, thereby reflecting the population’s genetic stability [51]. In the *A. polyzonata* populations examined, *He* values were consistently higher than Ho values, indicating potential mating among individuals with identical genotypes within the populations, leading to a certain degree of genetic similarity.

The Shannon index can, to some extent, reflect the level of genetic diversity in a species, with higher values corresponding to greater genetic diversity [18,28]. The mean Shannon index of the *A. polyzonata* populations in this study was 1.116. Compared with other snail species, such as *C. cathayensis* (1.513) [11] and *Babylonia areolata* (1.914) [52], the 12 *A. polyzonata* populations exhibited lower genetic diversity. It is speculated that inbreeding within *A. polyzonata* populations contributed to this reduced genetic diversity. Possible underlying causes are as follows: (1) A sharp decline in wild populations. For instance, the Luosifen industry relies heavily on harvesting wild resources, which may accelerate the depletion of snail populations [53]. (2) The invasion of *P. canaliculata*, which has occupied the ecological niche of native snails [54]. Currently, the analysis of the influence of these two factors is primarily based on speculative inferences derived from existing ecological knowledge and regional actual conditions, lacking direct quantitative data and statistical analysis support. Therefore, in the future, we will focus on collecting environmental data, such as fishing intensity and the distribution density of *P. canaliculata*, and analyze their correlations with genetic parameters through methods like Mantel tests in order to more rigorously reveal the driving factors influencing the genetic diversity of *A. polyzonata*. (3) *A. polyzonata* is primarily distributed in Southern Guangxi, including regions such as Beihai, Fangchenggang, Nanning, and Qinzhou, which lie south of the Tropic of Cancer and experience prolonged high-temperature periods [2]. Its germplasm resources have a relatively restricted distribution, and their restoration may be a slow process. Therefore, it is imperative to enhance awareness of *A. polyzonata* conservation in Guangxi to prevent the degradation of its germplasm resources.

In a specific ecological context, an in-depth investigation into genetic differentiation and population genetic structure is crucial for understanding the adaptability of organisms and the mechanisms underlying their persistence. The extent of gene exchange between populations is a key factor influencing the degree of genetic differentiation among them. Numerous studies have demonstrated that when gene flow (*Nm*) > 1, frequent gene exchange occurs between populations, resulting in relatively low genetic differentiation. This indicates that in such cases, the impact of gene flow on genetic differentiation between populations outweighs that of genetic drift. Conversely, when gene flow (*Nm*) < 1, the level of gene exchange between populations decreases significantly, and the degree of genetic differentiation increases, suggesting that genetic drift exerts a greater influence on genetic differentiation between populations under this scenario [55,56]. The *Nm* values among *A. polyzonata* populations range from 0.361 to 14.456, with most populations exhibiting *Nm* values less than 1. This suggests that in *A. polyzonata* populations, genetic drift and other stochastic factors have a more substantial impact on inter-population genetic differentiation than gene flow.

Genetic differentiation between populations is assessed using the fixation index (*Fst*). Specifically, *Fst* values between 0 and 0.05 indicate weak genetic differentiation, values between 0.05 and 0.15 indicate moderate differentiation, values between 0.15 and 0.25 indicate high differentiation, and values greater than 0.25 indicate extremely high genetic differentiation [57]. The degree of genetic differentiation varies among different *A. polyzonata* groups: differentiation between the TD and LA groups is relatively weak, as is that between the LC and LZ groups (*Fst* < 0.05). In contrast, the YH and HZ groups exhibit relatively high genetic differentiation from most other groups (*Fst* > 0.25). Nevertheless, despite the considerable geographical distance between the LC and LZ populations, their genetic differentiation is minimal, which may be attributed to “long-distance convergent evolution” driven by directed selection pressures [58]. Conversely, QN and YH populations are geographically close yet exhibit the highest level of genetic differentiation, which may be due to habitat fragmentation (artificial barriers) preventing gene flow between the two populations [59].

Results from molecular variance analysis (AMOVA) indicate that variation within *A. polyzonata* populations constitutes the primary source of total variation. This pattern is also observed in other Viviparidae snail species, such as *C. chinensis* [4] and *C. cathayensis* [11]. It further suggests a lack of individual migration or hybridization between these populations, meaning genetic variation cannot be homogenized through gene flow. Instead, divergent environmental selection pressures (climate and food resources) drive population differentiation, leading adaptive variations to be primarily concentrated among populations [60].

Genetic distance serves as an indicator of the genetic relationships among distinct biological populations [61]. The UPGMA dendrogram, which illustrates hierarchical clustering, reveals two primary clades, suggesting that the populations within these clades may share a relatively recent common ancestor while retaining a certain level of genetic diversity [62]. Furthermore, the two main branches of the UPGMA dendrogram do not represent the sampling geographical fitting pattern, but can be grouped according to the coastal water systems (QN, FC, ST, YH, and XD) and the inland water systems (LA, TD, LZ, YN, LC, LN, and HZ).

The results of principal coordinate analysis (PCoA) indicate that the distribution of the samples in the multi-dimensional space shows a certain similarity to the UPGMA clustering (samples such as LN, HZ, YN, and TD are clustered together). The clear separation of certain populations in the PCoA plot, such as YH and QN populations, indicates measurable genetic differentiation, whereas overlapping clusters reflect varying degrees of genetic connectivity between populations [63].

Structural analysis revealed that the optimal solution corresponds to K = 2, with significantly weaker support for more complex population structures. This suggests that *A. polyzonata* in the Guangxi region may comprise two distinct evolutionary lineages. The bar chart from the structural analysis provides a more intuitive illustration of this differentiation and highlights the varying compositional ratios between these two genetic clusters. We hypothesize that several factors may have contributed to the formation of this population structure: (1) Geographical barriers, such as the distribution of water systems and terrain in the Guangxi region, have restricted gene flow between populations [64], leading to the gradual accumulation of genetic differences. (2) Variations in habitat characteristics, including hydrological features, may have exerted selective pressures [65], driving genetic differentiation among populations. (3) Human activities, such as agricultural practices and urban construction, may have altered natural dispersal patterns [66,67]. Although this study, based on nine transcriptome-derived pairs of microsatellite markers, initially analyzed the genetic diversity and differentiation characteristics of different populations, further research is still needed to explore the influence of geographical micro-environments (altitude, water quality, and climate gradient) and temporal dynamics (breeding period and non-breeding period) on the genetic characteristics of the populations. Therefore, in the subsequent studies, we will build on this foundation to conduct a systematic improvement.

## 5. Conclusions

The findings of this study reveal that *A. polyzonata* is confronting a decline in genetic resources, characterized by low population genetic diversity, widespread inbreeding, significant heterozygote deficiency, high genetic differentiation among populations, and limited gene exchange. Therefore, measures ensuring the protection of its wild resources are of great significance. We should expand the breeding of this freshwater snail species to replace the use of wild resources. Fortunately, we discovered that the LA population of *A. polyzonata* has relatively high genetic quality, making it the preferred object for germplasm resource breeding in the Guangxi region and helping to establish a wild germplasm resource reserve. Overall, this study provides a scientific basis for the protection and sustainable utilization of *A. polyzonata* germplasm resources in the Guangxi region. However, in the future, it is necessary to combine mitochondrial genes and single-nucleotide polymorphisms to analyze their genetic diversity and genetic structure, and further optimize their protection strategy.

## Figures and Tables

**Table 1:** Information on sampling points of *Angulyagra polyzonata* in Guangxi.

Group	Sampling Points	Longitude and Latitude	Number/n
Yongning (YN)	Yongning District, Nanning City	108°31′32″ E, 22°39′19″ N	30
Tiandeng (TD)	Tiandeng County, Chongzuo City	107°17′45″ E, 23°14′32″ N	30
Long’an (LA)	Long’an County, Nanning City	107°47′18″ E, 23°8′56″ N	30
Longzhou (LZ)	Longzhou County, Chongzuo City	106°45′58″ E, 22°19′3″ N	30
Luchuan (LC)	Luchuan County, Yulin City	110°12′11″ E, 22°1′0″ N	30
Fangcheng (FC)	Fangcheng District, Fangchenggang City	108°31′26″ E, 21°55′44″ N	30
Qinnan (QN)	Qinnan District, Qinzhou City	108°44′35″ E, 21°44′16″ N	30
Yinhai (YH)	Yinhai District, Beihai City	109°16′20″ E, 21°27′47″ N	30
Xingdao (XD)	Xingdao Lake Town, Hepu County, Beihai City	109°7′40″ E, 21°45′29″ N	30
Shatian (ST)	Shatian Town, Hepu County, Beihai City	109°39′53″ E, 21°30′58″ N	30
Hezhou (HZ)	Babu District, Hezhou City	111°32′54″ E, 24°24′37″ N	30
Liannan (LN)	Liannan District, Liuzhou City	109°20′26″ E, 24°18′28″ N	30

**Table 2:** The number and distribution of SSR loci in *Angulyagra polyzonata*.

Category	Number
Total Number of Sequences Examined (n)	7,042,688
Total Size of Examined Sequences (bp)	1,657,747,000
Total Number of Identified SSRs (n)	798,244
Number of SSRs Present In Compound Formation (n)	126,494
The Number of Sequences Containing SSR Loci (n)	664,946
Number of Sequences Containing More Than One SSR (n)	108,342
Frequency of SSR Occurrence ^1^ (%)	11.33
Frequency of SSR Occurrence ^2^ (%)	9.44

Note: ^1^ the frequency of SSR loci occurrence in the detected total sequence; ^2^ the frequency of SSR sites appearing in the sequences containing SSR.

**Table 3:** The distribution of each repeat type of the *Angulyagra polyzonata* SSR loci.

Type of Repeating Unit	Number of Loci (n)	Percentage (%)	Average Length (bp)	SSR Motif Type Number
Mono-nucleotide	266,097	33.34	11.26	4
Di-nucleotide	380,290	47.64	17.67	6
Tri-nucleotide	74,729	9.36	19.62	20
Tetra-nucleotide	75,213	9.42	58.45	56
Penta-nucleotide	1604	0.20	56.37	82
Hexa-nucleotide	311	0.04	43.98	36

**Table 4:** Number of SSRs of different types and different quantities.

Repeat Number	Mono- Nucleotide	Di- Nucleotide	Tri- Nucleotide	Tetra- Nucleotide	Penta- Nucleotide	Hexa- Nucleotide
5	0	0	36,322	17,416	572	127
6	0	129,787	16,641	7368	122	72
7	0	81,165	7615	5003	83	59
8	0	51,198	4448	2929	74	7
9	0	30,314	3083	2212	31	0
10	129,027	19,702	1638	2019	46	1
11	60,970	13,713	1156	1541	38	0
12	31,304	9583	775	1623	36	0
13	17,927	8164	683	1855	42	2
14	10,486	6344	380	1438	86	1
15	6121	4773	375	1448	83	0
16	3331	3202	281	1356	3	20
17	2070	2646	183	1386	21	22
18	1260	1972	142	1829	41	0
19	906	1856	184	1753	48	0
20	689	1451	304	1682	5	0
>20	2006	14,420	519	22,355	273	0

**Table 5:** 9 Basic information regarding *Angulyagra polyzonata* primers.

Loci	Repeat Motif	Primer Sequence (5′-3′)	Size Range/bp	Fluorescence Labeling (5′)
DLJL042	(CAT)5	F: CCGAGAGTTCATAAGCAGCA	173–197	FAM
		R: TGTATTATGCAAGGCCCACA		
DLJL017	(ACTA)8	F:CCTAACCAACCAACTGACCG	124–158	FAM
		R: GTTTGCTTCTGCAATCTGGC		
DLJL025	(TAA)6	F: GCGCAACATTACATTGAACG	260–280	TAMRA
		R: ACGAGGAATGTCCATGAAGG		
DLJL126	(TGTC)8	F: AATTCTCGCGTACACTTGCC	226–262	FAM
		R: TGATATGACGCGTGGATGTT		
DLJL021	(TGTT)9	F:ACGGTCAGCAAAGCCTCTAA	154–208	HEX
		R: TCTTCACTTGTTGCCGTGAC		
DLJL112	(TACA)5	F: CGTGGGCCAGAGACATAGTT	212–252	TAMRA
		R: GAGTTCCAGTGATGCGTGTG		
DLJL033	(ATT)7	F: CGAAATTTGAGGCAGGAAAA	183–211	ROX
		R: GGAAGCTGTTACCTTCTGCG		
DLJL095	(GAT)7	F:AACCACCGCACTAGGTCAAG	277–319	HEX
		R: CCGCTAACGACGCCATACTA		
DLJL125	(ATCC)9	F:CGGCATAGTTTCAAACAGCA	224–272	ROX
		R: GATTTGTGTTCCAAAGCGGT		

**Table 6:** Genetic polymorphisms of 9 pairs of *Angulyagra polyzonata* microsatellite loci.

Locus	*Na*	*Ne*	*I*	*Ho*	*He*	*F*	*Hs*	*PIC*	Prob	Signif
DLJL017	10	3.848	1.649	0.484	0.74	0.346	0.87	0.702	0.000	***
DLJL021	12	4.739	1.76	0.417	0.789	0.472	0.894	0.759	0.000	***
DLJL025	9	3.658	1.591	0.474	0.727	0.348	0.863	0.697	0.000	***
DLJL033	11	6.285	2.043	0.537	0.841	0.362	0.92	0.823	0.000	***
DLJL042	5	3.388	1.391	0.356	0.705	0.496	0.852	0.662	0.000	***
DLJL095	24	7.856	2.371	0.42	0.873	0.519	0.936	0.861	0.000	***
DLJL112	15	3.788	1.732	0.494	0.736	0.328	0.868	0.71	0.000	***
DLJL125	18	7.495	2.277	0.598	0.867	0.310	0.933	0.853	0.000	***
DLJL126	15	5.121	1.987	0.537	0.805	0.332	0.902	0.783	0.000	***
Mean	13.222	5.131	1.867	0.480	0.787	0.390	0.893	0.761		
Stdev	5.563	1.701	0.325	0.074	0.063	0.081	0.032	0.073		

Note: *Na*: observed alleles; *Ne*: effective allele; *Ho*: observed heterozygosity; *I*: Shannon index; *He*: expected heterozygosity; *F*: the inbreeding coefficient, used to characterize the magnitude of deviation between actual and theoretical values; *HS*: Subpopulation Heterozygosity; *PIC*: polymorphic information index; Prob: Probability Value; Signif: significant (*** represents significant differences, *p* < 0.001).

**Table 7:** The genetic diversity of 12 *Angulyagra polyzonata* populations.

Populations	*Na*	*Ne*	*I*	*Ho*	*He*	*F*
YN	6.333	3.437	1.417	0.530	0.684	0.227
TD	6.111	3.921	1.464	0.599	0.723	0.173
LA	6.667	4.405	1.564	0.520	0.749	0.311
LZ	6.333	2.761	1.178	0.522	0.560	0.073
LC	5.444	2.498	1.093	0.414	0.536	0.273
FC	3.000	1.888	0.736	0.407	0.441	0.078
QN	4.778	1.869	0.795	0.373	0.390	0.028
YH	3.222	1.663	0.552	0.285	0.299	0.054
XD	5.556	3.041	1.230	0.518	0.627	0.177
ST	5.111	3.472	1.328	0.635	0.672	0.068
HZ	3.556	2.309	0.945	0.424	0.530	0.190
LN	4.333	2.757	1.093	0.530	0.598	0.126

**Table 8:** Gene flow (upper triangle) and fixation index (lower triangle) of 12 populations of *Angulyagra polyzonata*.

Population	YN	TD	LA	LZ	LC	FC	QN	YH	XD	ST	HZ	LN
YN	-	4.750	4.214	1.154	1.187	1.170	0.851	0.730	2.955	3.128	2.023	2.955
TD	0.050	-	14.456	1.395	1.428	1.139	0.886	0.846	3.271	2.875	1.734	2.591
LA	0.056	0.017	-	2.382	2.559	1.274	1.080	0.946	4.295	3.039	1.575	2.023
LZ	0.178	0.152	0.095	-	11.655	0.897	0.897	1.032	1.486	1.212	0.708	0.818
LC	0.174	0.149	0.089	0.021	-	0.861	0.902	0.982	1.658	1.332	0.742	0.823
FC	0.176	0.180	0.164	0.218	0.225	-	0.861	0.549	1.045	0.975	0.719	0.814
QN	0.227	0.220	0.188	0.218	0.217	0.225	-	0.361	0.775	1.059	0.597	0.666
YH	0.255	0.228	0.209	0.195	0.203	0.313	0.409	-	0.935	0.754	0.485	0.494
XD	0.078	0.071	0.055	0.144	0.131	0.193	0.244	0.211	-	2.915	1.428	1.474
ST	0.074	0.080	0.076	0.171	0.158	0.204	0.191	0.249	0.079	-	1.943	1.549
HZ	0.110	0.126	0.137	0.261	0.252	0.258	0.295	0.340	0.149	0.114	-	1.256
LN	0.078	0.088	0.110	0.234	0.233	0.235	0.273	0.336	0.145	0.139	0.166	-

**Table 9:** AMOVA results for 12 populations of *Angulyagra polyzonata*.

Source of Variation	Degree of Freedom	Total Variance	Variance of Mean	Estimated Difference Value	Mutation Percentage
Among Pops	11	685.560	62.324	0.984	27%
Within Pops	708	1902.700	5.395	2.697	73%
Total	719	2588.260	67.719	3.681	100%

**Table 10:** Genetic distance of 12 populations of *Angulyagra polyzonata*.

Population	YN	TD	LA	LZ	LC	FC	QN	YH	XD	ST	HZ	LN
YN		0.657	0.719	0.381	0.363	0.593	0.486	0.494	0.364	0.413	0.673	0.709
TD			0.328	0.570	0.648	0.523	0.614	0.691	0.440	0.394	0.303	0.258
LA				0.637	0.672	0.612	0.639	0.673	0.455	0.431	0.418	0.398
LZ					0.145	0.526	0.533	0.424	0.344	0.516	0.645	0.641
LC						0.523	0.488	0.381	0.372	0.516	0.630	0.629
FC							0.439	0.569	0.515	0.535	0.627	0.597
QN								0.652	0.558	0.467	0.684	0.664
YH									0.430	0.630	0.722	0.733
XD										0.349	0.511	0.526
ST											0.343	0.456
HZ												0.384
LN												

## Data Availability

The data supporting the reported results of this study can be provided from the corresponding author upon reasonable request.

## Supplementary Materials

The following supporting information can be downloaded at https://www.mdpi.com/article/10.3390/biology14101424/s1. Table S1: Statistics of initial sequencing data; Table S2: Statistical results of the FLASH integration sequence; Table S3: Statistics of SSR clustering results; Table S4: Statistics of SSR clustering results; Table S5: Original data statistics of Structure Analysis for 12 populations (K = 2); Figure S1: Photographs illustrating the morphology of *A. polyzonata*; Figure S2: Structure results of 360 samples of *Angulyagra polyzonata* (K = 2). The original sequencing materials were uploaded to http://www.ncbi.nlm.nih.gov/, with accession number PRJNA1310451.
