# Supplementary material for: Microsatellite Markers Developed Based on Transcriptomic Data Reveal the Genetic Diversity and Population Genetic Structure of Angulyagra polyzonata in Guangxi, China
Source: Biology (Basel). 2025 Oct 16;14(10):1424. doi: 10.3390/biology14101424 (PMC12562163; doi:10.3390/biology14101424)
Supplement: Supplementary file 1 [file biology-14-01424-s001.zip › biology-3829023-supplementary.pdf]

Table S1. Statistics of initial sequencing data

Sample	Reads_Num	Total_Bases(bp)	N(%)	GC(%)	Q20(%)	Q30(%)
Fangcheng(FC)-1	7,835,098	1,128,254,112	0.0	36.79	98.93	96.87
Yinhai(YH)-1	9,179,982	1,321,917,408	0.0	36.88	98.95	96.94
Shatian(ST)-1	11,841,374	1,705,157,856	0.0	36.7	98.95	96.96

Table S2. Statistical results of the FLASH integration sequence

Sample	Total Pairs	Combined Pairs	Uncombined Pairs	Percent Combined %
popA	14,166,848	7,042,688	7,124,160	49.71

Table S3. Statistics of SSR clustering results

Items	Count(popA)	Percentage(popA)
SSR-Containing Sequences	664,946	100%
SSR-Containing Sequences With Flanking Sequence Length ≥ 20 bp	281,408	42.32%
Clusters	14,867	2.23%

Table S4. Statistics of SSR clustering results

Iterms	Count of Clusters(popA)	Percentage(popA)
SSLP=1	9,575	64.40%
SSLP=2	2,700	18.16%
SSLP=3	1,158	7.78%
SSLP=4	611	4.10%
SSLP=5	357	2.40%
SSLP=6	209	1.40%
SSLP=7	128	0.86%
SSLP=8	72	0.48%
SSLP=9	26	0.17%
SSLP>=10	31	0.20%
Total	14,867	100%

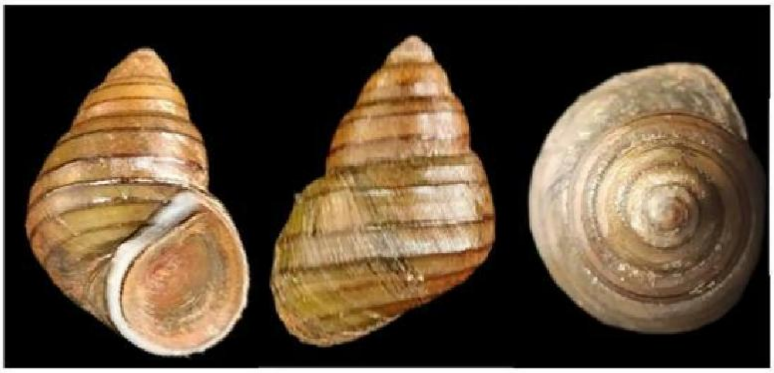

Figure S1.Photographs illustrating the morphology of *Angulyagra polyzonata*.

Note:Images from left toright show the front view,back view,and side view.

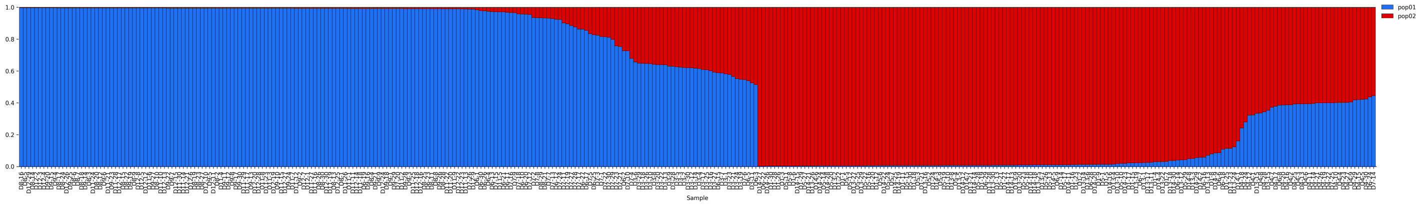

Figure S2. Structure results of 360 samples of *Angulyagra polyzonata* (K=2)

Table S5. Original data statistics of Structure Analysis for 12 populations (K =2)

pop	Sample	Cluster1	Cluster2
FC	D7-20	0.9922	0.0078
FC	D7-26	0.9883	0.0117
FC	D7-15	0.9719	0.0281
FC	D7-16	0.9669	0.0331
FC	D7-9	0.9662	0.0338
FC	D7-18	0.958	0.0421
FC	D7-12	0.9544	0.0456
FC	D7-2	0.9355	0.0645
FC	D7-8	0.9348	0.0652
FC	D7-7	0.9321	0.0678
FC	D7-13	0.9292	0.0709
FC	D7-24	0.9238	0.0762
FC	D7-11	0.9014	0.0986
FC	D7-19	0.8957	0.1044
FC	D7-27	0.8853	0.1147
FC	D7-28	0.8761	0.1239
FC	D7-17	0.8626	0.1374
FC	D7-5	0.8346	0.1653
FC	D7-3	0.8246	0.1754
FC	D7-1	0.8165	0.1835
FC	D7-22	0.815	0.1849
FC	D7-30	0.8113	0.1887
FC	D7-29	0.7983	0.2018
FC	D7-23	0.7575	0.2424
FC	D7-21	0.7533	0.2467
FC	D7-10	0.7259	0.2741
FC	D7-4	0.6788	0.3212
FC	D7-6	0.5459	0.4541
FC	D7-14	0.4456	0.5544
FC	D7-25	0.401	0.5991
HZ	D13-1	0.0257	0.9744
HZ	D13-26	0.0055	0.9946
HZ	D13-2	0.006	0.9939
HZ	D13-27	0.0064	0.9936
HZ	D13-29	0.0065	0.9935
HZ	D13-16	0.0072	0.9929
HZ	D13-5	0.0079	0.9921
HZ	D13-8	0.0079	0.9921
HZ	D13-21	0.008	0.992
HZ	D13-28	0.0083	0.9917
HZ	D13-30	0.0086	0.9914
HZ	D13-9	0.0091	0.9909

Table S5. (continued)

pop	Sample	Cluster1	Cluster2
HZ	D13-20	0.0097	0.9903
HZ	D13-25	0.011	0.989
HZ	D13-7	0.0113	0.9888
HZ	D13-14	0.0135	0.9866
HZ	D13-6	0.0134	0.9866
HZ	D13-4	0.0151	0.9849
HZ	D13-10	0.0189	0.9811
HZ	D13-19	0.0245	0.9755
HZ	D13-17	0.0261	0.9738
HZ	D13-11	0.0267	0.9733
HZ	D13-22	0.0317	0.9683
HZ	D13-18	0.038	0.962
HZ	D13-12	0.042	0.9581
HZ	D13-3	0.0508	0.9492
HZ	D13-13	0.071	0.929
HZ	D13-23	0.1147	0.8853
HZ	D13-24	0.1237	0.8763
HZ	D13-15	0.3343	0.6657
LA	D4-18	0.2416	0.7584
LA	D4-24	0.2797	0.7203
LA	D4-1	0.3231	0.6769
LA	D4-12	0.323	0.6769
LA	D4-8	0.3361	0.664
LA	D4-28	0.3437	0.6563
LA	D4-7	0.3725	0.6275
LA	D4-17	0.3786	0.6214
LA	D4-20	0.3864	0.6136
LA	D4-16	0.3867	0.6133
LA	D4-2	0.387	0.613
LA	D4-25	0.3924	0.6075
LA	D4-3	0.3932	0.6068
LA	D4-13	0.3939	0.606
LA	D4-6	0.3947	0.6053
LA	D4-11	0.3948	0.6052
LA	D4-14	0.3964	0.6036
LA	D4-21	0.3999	0.6001
LA	D4-26	0.4002	0.5998
LA	D4-19	0.4008	0.5992
LA	D4-22	0.4009	0.5991
LA	D4-10	0.4013	0.5987
LA	D4-23	0.4022	0.5978
LA	D4-4	0.4022	0.5978

Table S5. (continued)

pop	Sample	Cluster1	Cluster2
LA	D4-27	0.4024	0.5975
LA	D4-9	0.4057	0.5943
LA	D4-29	0.4183	0.5817
LA	D4-15	0.4207	0.5793
LA	D4-5	0.4221	0.5778
LA	D4-30	0.4235	0.5765
LC	D6-22	0.0039	0.9961
LC	D6-29	0.0083	0.9917
LC	D6-9	0.0116	0.9884
LC	D6-28	0.0135	0.9865
LC	D6-7	0.0247	0.9753
LC	D6-14	0.0408	0.9592
LC	D6-16	0.059	0.9411
LC	D6-6	0.0868	0.9132
LC	D6-18	0.1089	0.8911
LC	D6-8	0.3849	0.6151
LC	D6-24	0.4374	0.5626
LC	D6-3	0.5146	0.4854
LC	D6-5	0.5389	0.4611
LC	D6-15	0.5889	0.411
LC	D6-2	0.7272	0.2727
LC	D6-25	0.8283	0.1717
LC	D6-17	0.8547	0.1453
LC	D6-27	0.8624	0.1376
LC	D6-12	0.9244	0.0756
LC	D6-20	0.9563	0.0437
LC	D6-10	0.9574	0.0426
LC	D6-11	0.9684	0.0317
LC	D6-21	0.9706	0.0294
LC	D6-13	0.9724	0.0276
LC	D6-4	0.9743	0.0256
LC	D6-26	0.9772	0.0228
LC	D6-30	0.9793	0.0207
LC	D6-1	0.9839	0.0162
LC	D6-23	0.9917	0.0083
LC	D6-19	0.9928	0.0072
LN	D14-27	0.0036	0.9964
LN	D14-26	0.0042	0.9959
LN	D14-15	0.0047	0.9953
LN	D14-21	0.0052	0.9948
LN	D14-24	0.0058	0.9943
LN	D14-6	0.0057	0.9943

Table S5. (continued)

pop	Sample	Cluster1	Cluster2
LN	D14-28	0.006	0.994
LN	D14-22	0.0068	0.9933
LN	D14-19	0.0068	0.9932
LN	D14-25	0.0068	0.9932
LN	D14-3	0.0075	0.9925
LN	D14-12	0.0081	0.992
LN	D14-7	0.008	0.992
LN	D14-14	0.0081	0.9919
LN	D14-17	0.0088	0.9912
LN	D14-13	0.009	0.991
LN	D14-10	0.0101	0.9899
LN	D14-18	0.0105	0.9895
LN	D14-5	0.0115	0.9885
LN	D14-11	0.012	0.988
LN	D14-20	0.0138	0.9862
LN	D14-16	0.0145	0.9855
LN	D14-23	0.0202	0.9799
LN	D14-4	0.0303	0.9697
LN	D14-30	0.0381	0.9619
LN	D14-8	0.0494	0.9506
LN	D14-29	0.0563	0.9437
LN	D14-2	0.0582	0.9418
LN	D14-9	0.085	0.915
LN	D14-1	0.1606	0.8394
LZ	D5-11	0.0047	0.9953
LZ	D5-8	0.0048	0.9953
LZ	D5-27	0.0052	0.9948
LZ	D5-14	0.006	0.994
LZ	D5-20	0.006	0.994
LZ	D5-5	0.0062	0.9938
LZ	D5-12	0.0063	0.9937
LZ	D5-25	0.0064	0.9936
LZ	D5-22	0.0065	0.9935
LZ	D5-16	0.0067	0.9934
LZ	D5-24	0.0067	0.9933
LZ	D5-6	0.0067	0.9933
LZ	D5-7	0.0067	0.9933
LZ	D5-15	0.0069	0.9931
LZ	D5-3	0.007	0.993
LZ	D5-30	0.007	0.993
LZ	D5-2	0.0071	0.9929
LZ	D5-9	0.0075	0.9925

Table S5. (continued)

pop	Sample	Cluster1	Cluster2
LZ	D5-19	0.0078	0.9923
LZ	D5-4	0.0079	0.9921
LZ	D5-28	0.0081	0.9919
LZ	D5-21	0.0087	0.9913
LZ	D5-23	0.0097	0.9903
LZ	D5-18	0.0101	0.9899
LZ	D5-26	0.0113	0.9888
LZ	D5-13	0.0113	0.9887
LZ	D5-17	0.0119	0.9881
LZ	D5-1	0.0144	0.9857
LZ	D5-10	0.0195	0.9805
LZ	D5-29	0.0435	0.9566
QN	D8-16	0.9963	0.0037
QN	D8-5	0.9961	0.0039
QN	D8-14	0.9959	0.0042
QN	D8-17	0.9958	0.0042
QN	D8-24	0.9958	0.0043
QN	D8-6	0.9957	0.0043
QN	D8-18	0.9956	0.0044
QN	D8-7	0.9956	0.0044
QN	D8-3	0.9955	0.0045
QN	D8-12	0.9954	0.0046
QN	D8-20	0.9954	0.0046
QN	D8-15	0.9953	0.0047
QN	D8-22	0.9953	0.0047
QN	D8-9	0.9952	0.0048
QN	D8-10	0.995	0.005
QN	D8-19	0.9948	0.0052
QN	D8-23	0.9948	0.0052
QN	D8-27	0.9948	0.0052
QN	D8-2	0.9946	0.0055
QN	D8-26	0.9938	0.0062
QN	D8-30	0.9935	0.0065
QN	D8-13	0.9933	0.0067
QN	D8-1	0.9932	0.0068
QN	D8-4	0.9925	0.0076
QN	D8-25	0.9919	0.0081
QN	D8-11	0.9917	0.0084
QN	D8-8	0.9914	0.0087
QN	D8-28	0.9911	0.0089
QN	D8-29	0.9331	0.0669
QN	D8-21	0.9323	0.0678

Table S5. (continued)

pop	Sample	Cluster1	Cluster2
ST	D12-2	0.9959	0.0042
ST	D12-29	0.9959	0.0042
ST	D12-3	0.9959	0.0042
ST	D12-4	0.9959	0.0042
ST	D12-26	0.9957	0.0043
ST	D12-17	0.9953	0.0047
ST	D12-12	0.9952	0.0048
ST	D12-5	0.9952	0.0048
ST	D12-8	0.9952	0.0048
ST	D12-13	0.995	0.005
ST	D12-10	0.9948	0.0052
ST	D12-15	0.9948	0.0052
ST	D12-21	0.9948	0.0052
ST	D12-6	0.9948	0.0052
ST	D12-11	0.9947	0.0053
ST	D12-20	0.9944	0.0056
ST	D12-28	0.9943	0.0057
ST	D12-19	0.9942	0.0058
ST	D12-27	0.9941	0.0059
ST	D12-1	0.9938	0.0062
ST	D12-7	0.9938	0.0062
ST	D12-30	0.9935	0.0064
ST	D12-25	0.9932	0.0068
ST	D12-14	0.9929	0.0071
ST	D12-24	0.9925	0.0075
ST	D12-16	0.9919	0.0081
ST	D12-18	0.9919	0.0081
ST	D12-22	0.9907	0.0094
ST	D12-23	0.9899	0.01
ST	D12-9	0.9881	0.0119
TD	D3-6	0.656	0.344
TD	D3-29	0.6493	0.3507
TD	D3-18	0.6477	0.3522
TD	D3-26	0.6474	0.3526
TD	D3-10	0.6463	0.3537
TD	D3-8	0.6415	0.3585
TD	D3-20	0.6399	0.3601
TD	D3-22	0.6396	0.3605
TD	D3-13	0.639	0.361
TD	D3-9	0.6299	0.3701
TD	D3-28	0.6298	0.3701
TD	D3-5	0.6261	0.3739

Table S5. (continued)

pop	Sample	Cluster1	Cluster2
TD	D3-3	0.6244	0.3756
TD	D3-2	0.6214	0.3786
TD	D3-30	0.6209	0.3791
TD	D3-12	0.6203	0.3797
TD	D3-14	0.6167	0.3833
TD	D3-4	0.6155	0.3845
TD	D3-17	0.6089	0.3911
TD	D3-25	0.608	0.3919
TD	D3-16	0.6015	0.3984
TD	D3-27	0.5923	0.4077
TD	D3-7	0.5868	0.4132
TD	D3-1	0.582	0.418
TD	D3-23	0.5781	0.4219
TD	D3-21	0.5651	0.4349
TD	D3-24	0.5513	0.4487
TD	D3-19	0.5481	0.4519
TD	D3-11	0.5251	0.475
TD	D3-15	0.3534	0.6466
XD	D11-24	0.9954	0.0046
XD	D11-21	0.9953	0.0048
XD	D11-28	0.9953	0.0047
XD	D11-6	0.9951	0.0049
XD	D11-10	0.995	0.005
XD	D11-14	0.9948	0.0052
XD	D11-30	0.9948	0.0052
XD	D11-4	0.9946	0.0054
XD	D11-17	0.9944	0.0057
XD	D11-29	0.9944	0.0056
XD	D11-8	0.9943	0.0057
XD	D11-23	0.9942	0.0058
XD	D11-3	0.9942	0.0058
XD	D11-15	0.9941	0.0059
XD	D11-7	0.9941	0.0059
XD	D11-12	0.994	0.0059
XD	D11-16	0.9938	0.0062
XD	D11-27	0.9938	0.0062
XD	D11-19	0.9934	0.0066
XD	D11-26	0.9931	0.0069
XD	D11-1	0.993	0.007
XD	D11-11	0.9929	0.0071
XD	D11-18	0.9928	0.0072
XD	D11-2	0.9922	0.0078

Table S5. (continued)

pop	Sample	Cluster1	Cluster2
XD	D11-25	0.9919	0.0081
XD	D11-20	0.991	0.009
XD	D11-22	0.991	0.009
XD	D11-9	0.9908	0.0093
XD	D11-13	0.9887	0.0114
XD	D11-5	0.9707	0.0293
YH	D9-15	0.9959	0.0042
YH	D9-20	0.9959	0.0042
YH	D9-4	0.9959	0.0042
YH	D9-25	0.9957	0.0043
YH	D9-14	0.9955	0.0045
YH	D9-6	0.9954	0.0046
YH	D9-23	0.9953	0.0047
YH	D9-19	0.9952	0.0048
YH	D9-27	0.9951	0.0049
YH	D9-22	0.9949	0.0051
YH	D9-7	0.9949	0.0051
YH	D9-5	0.9947	0.0054
YH	D9-11	0.9945	0.0055
YH	D9-12	0.9945	0.0055
YH	D9-17	0.9945	0.0055
YH	D9-30	0.9945	0.0055
YH	D9-8	0.9945	0.0055
YH	D9-13	0.9944	0.0056
YH	D9-10	0.9942	0.0058
YH	D9-24	0.9941	0.0059
YH	D9-2	0.9939	0.0061
YH	D9-16	0.9927	0.0073
YH	D9-21	0.9925	0.0075
YH	D9-9	0.9925	0.0075
YH	D9-1	0.9924	0.0077
YH	D9-18	0.9924	0.0076
YH	D9-29	0.9922	0.0078
YH	D9-28	0.9921	0.0079
YH	D9-3	0.9921	0.0079
YH	D9-26	0.9913	0.0087
YN	D1-28	0.0041	0.9959
YN	D1-30	0.0042	0.9959
YN	D1-26	0.0043	0.9957
YN	D1-3	0.0045	0.9955
YN	D1-6	0.0047	0.9953
YN	D1-29	0.0052	0.9948

Table S5. (continued)

pop	Sample	Cluster1	Cluster2
YN	D1-1	0.006	0.9939
YN	D1-22	0.0061	0.9939
YN	D1-20	0.0066	0.9934
YN	D1-12	0.0067	0.9933
YN	D1-11	0.0069	0.9931
YN	D1-18	0.007	0.993
YN	D1-21	0.0071	0.9929
YN	D1-24	0.0074	0.9926
YN	D1-10	0.0078	0.9922
YN	D1-19	0.0082	0.9918
YN	D1-13	0.0087	0.9913
YN	D1-27	0.0088	0.9912
YN	D1-14	0.0117	0.9883
YN	D1-16	0.0128	0.9872
YN	D1-23	0.0129	0.9871
YN	D1-2	0.0131	0.9869
YN	D1-9	0.0143	0.9858
YN	D1-5	0.0145	0.9856
YN	D1-15	0.0234	0.9766
YN	D1-17	0.0234	0.9766
YN	D1-4	0.0303	0.9698
YN	D1-7	0.0326	0.9675
YN	D1-8	0.0785	0.9215
YN	D1-25	0.1141	0.886
